# ADGRG1 Is a Predictor of Chemoresistance and Poor Survival in Cervical Squamous Carcinoma

**DOI:** 10.3389/fonc.2021.671895

**Published:** 2021-07-22

**Authors:** Shuo Zhang, Kui Guo, Ying Liang, Kun Wang, Shuyan Liu, Xingsheng Yang

**Affiliations:** Department of Obstetrics and Gynecology, Qilu Hospital of Shandong University, Jinan, China

**Keywords:** ADGRG1, cisplatin resistance, proliferation, PI3K/Akt/mTOR, cervical cancer

## Abstract

**Background:**

Cisplatin is the first-line chemotherapy for cervical cancer. Cisplatin resistance has always been one of the most significant barriers to acquiring better outcomes. However, the complex molecular mechanisms accounting for the phenomenon are not completely clear.

**Methods:**

Construction of the cisplatin-resistant cell model of cervical cancer, then performing RNA sequencing and bioinformatic analysis of the differential expression genes. Then Adhesion G protein-coupled receptor G1 (ADGRG1) was screened out as our target gene. Gene Expression Profiling Interactive Analysis (GEPIA) was searched to show the expression level of ADGRG1 in cervical cancer and normal tissue. Kaplan-Meier Plotter (Kmplot) was used to explore the relationship of its expression with survival data. Tissue specimens were used to verify the relationship between the clinicopathological characteristics and ADGRG1 expression. Then we explored the roles of ADGRG1 in tumorigenesis through *in vitro* and *in vivo* assays.

**Results:**

We found the ADGRG1 was significantly overexpressed in cervical cancer tissues compared to corresponding normal tissues. Higher ADGRG1 expression was correlated with poor progress-free survival. Knockdown of ADGRG1 markedly suppressed cell proliferation, migration, and invasion and increased cell sensitivity to cisplatin *in vitro*. Similarly, the role of ADGRG1 knockdown on tumorigenicity and sensitivity to cisplatin treatment was verified *in vivo*. The underlying mechanism was explored by western blotting that ADGRG1 knockdown inhibited tumorigenesis by PI3K/Akt/mTOR signaling pathway.

**Conclusion:**

ADGRG1 acts as an oncogene to maintain tumorigenicity, migration, and invasion, and its depressed expression prompts sensitivity to cisplatin. Thus, ADGRG1 may represent a potential prognostic marker and possible therapeutic target for cervical cancer.

## Introduction

Cervical cancer is the fourth most cancer in women with an age-standardized incidence of 13.1 per 100,000 women globally (1). Although preventive and screening measures for cervical cancer have greatly developed, the prognosis of advanced cases is far from satisfactory (2). For early-stage cases, surgery is generally preferred and curative. However, the overall response rate among radiotherapy and chemotherapy is not high in patients (3), which may be related to resistance to chemoradiation. Cisplatin has been considered the primary chemotherapy for metastatic or recurrent cases, even in combination with radiotherapy as a radiosensitizer for locally advanced cervical cancer (4–6). A phase III randomized trial by Yoichi Aoki concluded that there was an approximate 30% overall response rate to cisplatin in patients with stage IVB, recurrent, or persistent cervical cancer (7). Therefore, overcoming cisplatin resistance is of great importance to achieve a higher response rate and better prognosis (8).

G Protein-Coupled Receptors (GPCRs) are proteins encoded by more than 800 genes in the human genome, and they are further classified into five subfamilies including Glutamate, Rhodopsin, Adhesion, Frizzled, and Secretin (9). In recent years, multiple adhesion GPCRs (aGPCRs) have been involved in a variety of progression of physiological functions, genetic disorders, and tumorigenesis (10).

ADGRG1, also called G-protein-coupled receptor 56 (GPR56), is an important member of the aGPCR family. There has been a lot of evidence that ADGRG1 participates in a variety of biological processes, including brain development (11, 12), hematopoietic stem cell generation (13), male fertility (14), myoblast fusion (15), muscle hypertrophy (16), tumorigenesis (17–22). However, the clinical significance and potential possible mechanism of ADGRG1 in regulating tumorigenesis of cervical cancer remain unclear. Therefore, we designed experiments to explore the expression level and biological function of ADGRG1 in cervical squamous cancer.

## Material and Methods

### Cell Culture and Construction of Stable Cisplatin−Resistant Cell Line

Siha, which was a human cervical squamous carcinoma cell line, was purchased from Procell Life Science & Technology Co and cultured in α-MEM medium (BI; Biological Industries) supplemented with 10% fetal bovine serum (FBS; BI) and 1% penicillin-streptomycin (Solarbio). And Siha cells grow at 37°C with 5% carbon dioxide in a humidified environment. The resistant cell line was developed by dose-escalation continuous exposure to cisplatin (Selleck) from the parental cell line (Siha-N). Siha was cultured in α-MEM medium supplemented with cisplatin started with a concentration at 0.125 μg/ml. When the concentration of cisplatin reached 3 μg/ml and the cells can grow stably at this concentration, a stable cisplatin-resistant Siha called Siha-DDP was established. The viability rates and IC50 values in cisplatin were detected by Cell Counting Kit‐8 (CCK8) assay.

### CCK8 Assay

The parental cell line (Siha-N) and cisplatin-resistant cell line (Siha-DDP) were respectively seeded in 96-well plates at an initial density of 1×10^4^ cells/well in 100 μl α-MEM medium per well. Cisplatin at gradient concentration (0, 2, 4, 8, 16, 32, and 48 μg/ml) was added later after cell attachment. After 48 h of exposure to cisplatin, cell viability was assessed by Cell Counting Kit‐8 (CCK‐8; Dojindo Laboratories). Ten μl CCK‐8 reagent and 90 μl medium were added to each well and incubated for another 1 h in the cell incubator. The absorbance was measured at 450 nm wavelength. Six wells were used for repetition at each concentration. Cell viability rate curves were generated, and 50% inhibiting concentration (IC50) was obtained.

Similarly, Siha-N and Siha-DDP were respectively seeded in 96-well plates at a density of 1×10^3^ cells/well following the steps above to obtain the OD value. The values were measured every 24 h for continuous 6 days to plot the cell proliferation curve.

### Cell Apoptosis Detected by Flow Cytometry

Siha-NC (negative control) and Siha-KD (knockdown) were plated in six-well plates, respectively. Medium with and without cisplatin was added to the wells for another 24 h after cell attachment. Then cells were collected for flow cytometry to detect apoptosis. Cell apoptosis was determined by Annexin V-APC/7AAD apoptosis detection kit (BD Biosciences) following the manufacturer’s product instruction manual.

### RNA-Sequencing and Identification of Differentially Expressed Genes

The cisplatin-resistant cell lines (Siha-DDP1, Siha-DDP2, Siha-DDP3, Siha-DDP4, Siha-DDP5) were used as the test group, and the parental cell lines (Siha-N1, Siha-N2, Siha-N3) were regarded as the control group. Afterwards, the eight samples were sent to Annoroad Gene Technology performing RNA sequencing. Genes meeting the criterion of |log2Foldchange| ≥ 1 and p-value < 0.05 were identified as differentially expressed genes (DEGs). GEO datasets (https://www.ncbi.nlm.nih.gov/gds/) were searched for cisplatin-resistant cervical cancer. Finally, the overlap of the gene sets was obtained by Funrich software.

### GEPIA Analysis

We obtained the expression profile of ADGRG1 in cervical cancer and normal tissues by the GEPIA website (http://gepia.cancer-pku.cn/). The result was presented in the form of box plots.

### Survival Analysis of ADGRG1

The Kaplan-Meier plotter (http://kmplot.com/analysis/) was used to assess the survival of 174 cervical squamous cancer samples. Firstly, enter the gene “ADGRG1” and select the option “Auto select best cutoff.” After automatic calculation, the best performing threshold was used as a cutoff value between the lower and upper quartiles. The cervical cancer patients with FIGO stage I-IV were divided into high- and low-expression groups, followed by evaluating the Relapse-Free Survival using a Kaplan-Meier plot. The hazard ratio (HR) and log-rank P value were obtained from the webpage.

### Human Tissue Specimens

A total of 58 pairs of fresh cervical squamous carcinoma tissues and corresponding para-carcinoma normal tissues were obtained from patients with cervical carcinoma who underwent a radical hysterectomy at the Department of Obstetrics and Gynecology, Qilu Hospital, from 2018 to 2019. Proteins extracted from these fresh tissues were used for Western blotting.

Tissue slices derived from 132 cervical squamous cancer patients who underwent radical hysterectomy from January 2009 to December 2013 in Qilu Hospital were collected from the department of pathology.

All patients included in our study were histopathologically diagnosed with cervical squamous cell carcinoma, clinically staged FIGO I-II. None of these patients had a history of prior radiation or chemotherapy. Patients with other malignancies were excluded. Agreements for the following studies were approved by the Ethics Committee of Qilu Hospital of Shandong University. The patients and their families were informed of specimen collection, and the informed consent forms were signed.

### Western Blotting

Total protein of tissues and cells were lysed in a mixture of RIPA, protease inhibitor, and phosphatase inhibitors (100:1:1, Beyotime Institute of Biotechnology). BCA Protein Assay Kit (Beyotime Institute of Biotechnology) was used to detect protein concentrations. Thirty μg of protein samples were separated by 10% SDS-PAGE and then transferred to PVDF membranes (EMD Millipore). After being blocked with 5% fat-free milk for 1 h, the membranes were incubated with primary antibodies against ADGRG1 (1:1,000; Santa Cruz), mTOR (1:1,000; CST, Cell Signaling Technology), p-mTOR (1:1,000; CST), PI3K (1:1,000; CST), AKT (1:1,000; CST), p-AKT (1:2,000; CST), GAPDH (1:3,000; Servicebio) at 4°C overnight. After incubation with primary antibodies, the membranes were incubated in anti-rabbit or anti-mouse secondary antibodies (Cell Signaling Technology) for 1 h. Protein signals were visualized by the ECL detection system (Amersham Imager 600; GE).

### Immunohistochemistry

After baking for 1 h at 65°C, the paraffin‐embedded sections were deparaffinized in xylene and rehydrated in fractionated ethanol solutions. The slices were heated in citrate buffer (0.01 M, pH 6.0) for antigen retrieval. Then, 3% hydrogen peroxide was added to slices to block peroxidase. Sections were incubated with primary antibody (anti‐ADGRG1; 1:400; Affinity) at 4°C overnight. After washing with phosphate‐buffered saline (PBS), the sections were incubated with reagents 2 and 3 (PV‐9001; Zhong Shan Biotech Co Ltd) in sequence. Diaminobenzidine (DAB) was added to show the positive area. The total score of each slice was calculated by the method based on the percentage of positive cells and staining intensity (immunoreactive score = staining intensity × percentage of positive cells). Staining intensity was graded as 0 (negative), 1 (weak), 2 (moderate), 3 (strong). The percentage of positive cells was scored as 0 (<10%), 1 (10–30%), 2 (30–50%), 3 (50–70%), 4 (>70%). Cases with immunoreactive score ≤6 were defined as low-expression group, and those with score >6 were defined as high-expression group.

### Real-Time PCR

Total RNA from cells was extracted by Trizol reagent (Thermo Fisher Scientific) according to the manufacturer’s instructions. After detecting the concentration of mRNA, 10 μl of each sample was reverse-transcribed to cDNA using ReverTra Ace qPCR RT Master Mix with genomic DNA Remover (Toyobo Co). Quantitative real‐time polymerase chain reaction (qRT‐PCR) was performed using the SYBR Green (Toyobo Co). The relative mRNA expression was normalized to GAPDH and calculated using 2^−ΔΔCt^ calculation method.

### Si-RNA and Lentivirus Transfection

ADGRG1−targeting small interfering RNAs (si-RNAs) and negative control siRNAs (si−NC) were designed and synthesized by GenePharmaCorp. The ADGRG−siRNA sequences were as follows: siRNA-1428, 5’−GCAACCACUUGACCUACUUTT−3’; siRNA-1900, 5’−GGUGGAUGUGGACAACUAUTT−3’; siRNA-2178, 5’−CCUUUGCUUCUGGCACCU UTT−3’. The si−NC sequence was as follows: 5’−UUCUCCGAACGUGUCACGUTT−3’ (sense) and 5’−ACGUGACACGUUCGGAGA ATT−3’ (antisense). The si−RNAs and si−NC were transfected into Siha by INTERFERin (Polyplus Transfection) according to the manufacturer’s protocols. After 48 h, knockdown efficiency was evaluated by PCR and Western blotting. Lentiviruses carrying short hairpin RNA (shRNA: 5′‐GCAACCACTTGACCTACTT-3′) targeting ADGRG1 and negative control (shNC: 5′‐TTCTCCGAACGTGTCACGT-3′) were also synthesized by GenePharmaCorp.

### Immunofluorescence Assay

Cells cultured on sterile coverslips were fixed with 4% paraformaldehyde for 30 min. Following permeabilization with 0.4% Triton X-100 for 15 min, goat serum was used for blocking at room temperature for 30 min. The cells were incubated with primary anti-ADGRG1 antibody (1:400; Affinity) overnight at 4°C. After washing with PBS, the cells were stained with DyLight594 conjugate goat antirabbit immunoglobulin G (1:100; Abbkine Scientific) in the dark for 1 h. Subsequently, nuclei were stained with 4′,6‐diamidino‐2‐phenylindole (DAPI; Boster Biological Technology). Images were captured with a fluorescence microscope.

### Cell Migration and Invasion Assay

Wound scratch assay was conducted to evaluate cell migration. First, 80×10^4^ cells were planted on a six-well plate. After cell attachment at about 80–90% density, wounds were scratched by 200 μl pipette tips. Then replace the medium with the serum-free α-MEM medium. Take photos at 0, 24, 48, 72 h. Cell invasion ability was assessed by the transwell assay. Following coating the upper-side membrane of the transwell chamber (Corning Incorporate) with 60 μl Matrigel (1:7; Corning Incorporate), the upper side of the inserts were filled with 200 ul serum-free medium containing 10 × 10^4^ cells, and the lower chamber was filled with 600 μl medium containing 10% FBS. After being cultured for 24 h, invading cells were fixed with methanol for 15 min and stained with 0.1% crystal violet for 30 min. The number of invasive cells was counted in five random views (200×).

### Colony Formation Assay

One thousand cells per well were cultured in a six-well plate. Replace the medium with a cisplatin concentration of 2 ug/ml. After a total of 10 days of culture, the colonies were fixed with 100% methanol for 15 min and stained with 0.1% crystal violet for 30 min, then the number of colonies was counted.

### ADGRG1 Regulates Xenograft Growth and Sensitivity to Cisplatin Treatment *In Vivo*

The BALB/c nude mice (female, aged 5 weeks, 16.2 ± 2.6 g) were purchased from Gempharmatech Co and fed in SPF breeding units. Each mouse received subcutaneous inoculating of 8 × 10^6^ cell suspension bilaterally. The details were as follows: Siha-NC was injected into the right axilla, and Siha-KD was injected into the left axilla. After 10 days of inoculation, the mice were randomly divided into two groups (non-cisplatin treatment group, cisplatin treatment group, n = 6 per group). Cisplatin (2 mg/kg) and equal volume NS were injected intraperitoneally into nude mice every 5 days. Simultaneously, tumor volume (volume = width^2^ × length/2) was determined by vernier caliper and recorded. All mice were euthanized on day 25, and the tumors were completely excised and stored at −80°C.

### Statistics Analysis

The data were presented as means ± SD, and significant differences were determined using GraphPad 8.0. Student’s t-test and one-way ANOVA were used to determine statistical differences between groups. The chi-square test was used to analyze relationships between ADGRG1 expression and clinicopathological characteristics. P values < 0.05 were regarded as statistically significant.

## Results

### Construction of Cisplatin-Resistant Cell Line and Identification of Differentially Expressed Genes

**Figure 1:**
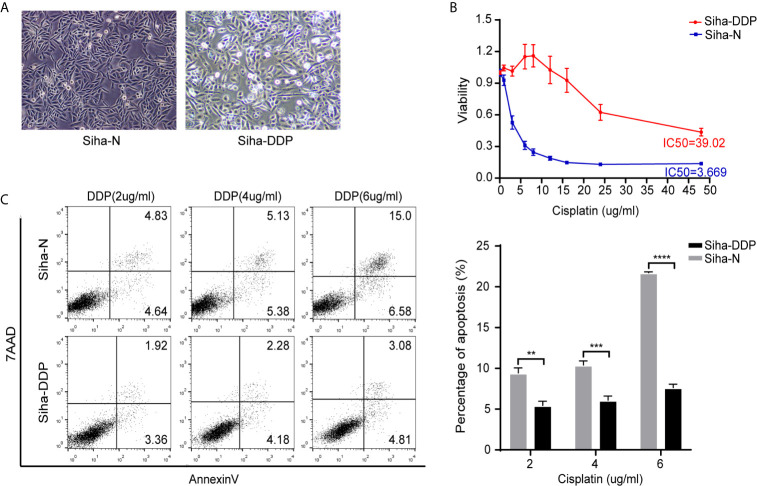
Construction of cisplatin-resistant Siha cell line. **(A)** Morphology of Siha-N and Siha-DDP. (Magnifications, 200×). **(B)** Cell viability under the increasing concentration of cisplatin detected by CCK8 assay. IC50, 50% inhibiting concentration. The data are presented as mean ± SD. Error bars indicate SD. **(C)** Cell apoptosis rate under various concentrations of cisplatin. The apoptosis rate = early apoptosis rate + late apoptosis rate. The data are presented as means ± SD of three independent experiments. Error bars indicate SD (**P < 0.01, ***P < 0.001, ****P < 0.0001).

**Figure 2:**
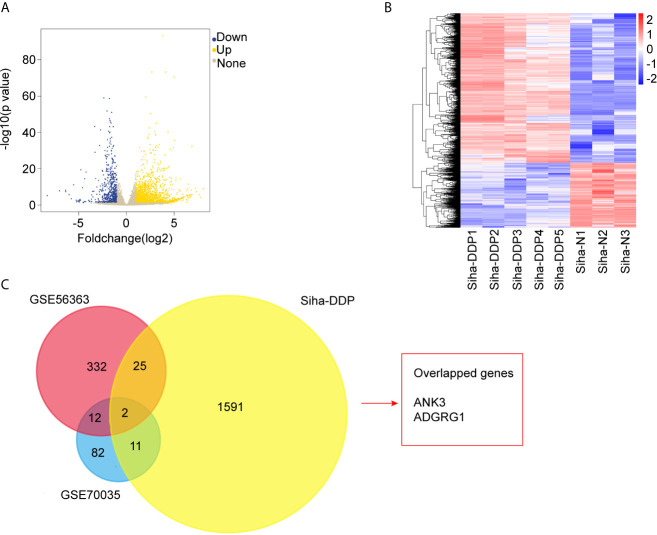
RNA-sequencing and bioinformatics analysis of differentially expressed genes (DEGs). **(A)** Volcano plots of the 32,808 expressed genes. The X-axis represents “log2 (Foldchange),” and the y-axis represents “-log10 (p-value).” Yellow color represented upregulated genes, and blue color represented downregulated genes in cisplatin-resistant Siha cells (Siha-DDP) compared with the parental cells (Siha-N). **(B)** Clustering heatmap of the 2,337 differential expressed genes (DEGs). The criteria for DEGs are |Log2Foldchange|>1 and adjp-value<0.05. **(C)** Overlapped genes of three datasets.

We developed a stable, resistant cervical cancer cell line to cisplatin called Siha-DDP following gradient concentration induction procedure. Having experienced long-term stimulation of cisplatin, Siha became polymorphic, especially much slender with increased intercellular space (**Figure 1A**). The cellular viability of Siha-DDP was significantly higher than the parental cell line Siha-N determined by CCK8 assay (**Figure 1B**). The IC50 of Siha-DDP and Siha-N were 39.02 and 3.669 μg/ml, respectively. So the resistance index (RI) was 10.635. Meanwhile, flow cytometry showed the apoptosis rate of Siha-DDP in cisplatin was significantly lower than Siha-N (**Figure 1C**). RNA sequencing was performed on cisplatin-resistant cell lines and cisplatin-sensitive cell lines (**Figure 2A**). A total of 2,337 DEGs were filtered out according to the criteria of |log2 Fold change|≥1 and padj < 0.05 (**Figure 2B**). The DEGs are composed of 1,629 upregulated genes and 708 downregulated genes. We searched GEO datasets (https://www.ncbi.nlm.nih.gov/gds/) using “cervical cancer” as the keyword. GSE56363 included 21 patients with locally advanced squamous cell carcinoma who were treated by chemoradiotherapy sensitized with cisplatin. A microarray experiment was performed to measure differences in gene expression between cervical cancer samples at a 6-month complete response (12 patients) and non-complete response (9 patients). In the datasets of GSE70035, all patients with locally advanced squamous cervical cancer received neoadjuvant chemotherapy using platinum and irinotecan followed by radical hysterectomy. Similarly, the GSE70035 showed gene expression profiles on six samples of neoadjuvant (NAC) responder and six samples of non-responder in cervical cancer. Then the two databases were analyzed respectively to obtain DEGs in the method of GEO2R. To find DEGs related to cisplatin resistance with the same expression trend in tissue microarray and cell RNA-sequencing, we used FunRich3.1.3 software to obtain the intersection of these three groups of DEGs (**Figure 2C**). As a result, ADGRG1 (GPR56) was screened out.

### ADGRG1 Was Overexpressed in Cervical Cancer, and Its Expression Level Was Negatively Correlated With a Poor Prognosis

**Figure 3:**
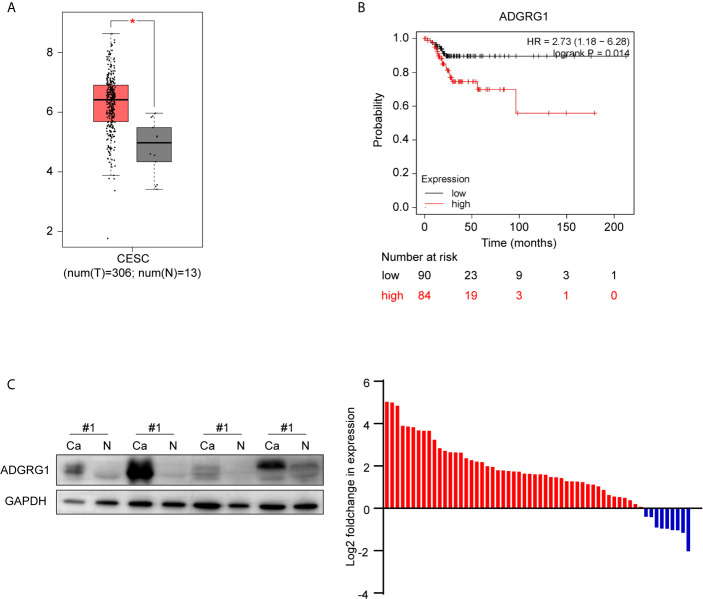
ADGRG1 expression in tissue and its correlation with survival. **(A)** ADGRG1 is overexpressed in cervical carcinoma tissues in the dataset from GEPIA (*p < 0.01). **(B)** Kaplan-Meier survival curves demonstrate that high expression of ADGRG1 was negatively correlated to relapse-free survival (RFS). **(C)** ADGRG1 protein expression in 58 pairs of cervical cancer tissues and adjacent normal cervix were detected by western blotting. Using GAPDH as an internal reference, ADGRG1 relative expression level was presented as log2(foldchange Ca/N). Red color represents overexpression in cancer tissue, and blue represents underexpression in cancer tissue.

We investigated the available dataset of ADGRG1 expression profile in human cervical cancer from GEPIA. We found that ADGRG1 was overexpressed in cervical carcinoma compared to normal cervix uteri (**Figure 3A**). To further evaluate the relationship between ADGRG1 expression levels and the prognosis of cervical cancer, survival statistics were generated from the Kaplan-Meier plotter. As shown in **Figure 3B**, survival data of 174 cervical cancer patients were followed up, and hazard ratios (HR) and p-values for statistical significance were determined. Poor replase-free survival probability was dramatically associated with a high expression of ADGRG1 (P = 0.014) (**Figure 3B**). So, high expression of ADGRG1 predicts poor prognosis in cervical cancer. To validate the expression of ADGRG1, we evaluated its protein levels of 58 paired primary tissues by western blotting. Among them, 84.5% (49 of 58) of cervical cancer specimens showed significant overexpression of ADGRG1 protein (**Figure 3C**). Overall, the results confirmed that ADGRG1 contributes to tumorigenesis and progression in cervical cancer.

### Correlation Between ADGRG1 Expression and Clinicopathological Factors

**Figure 4:**
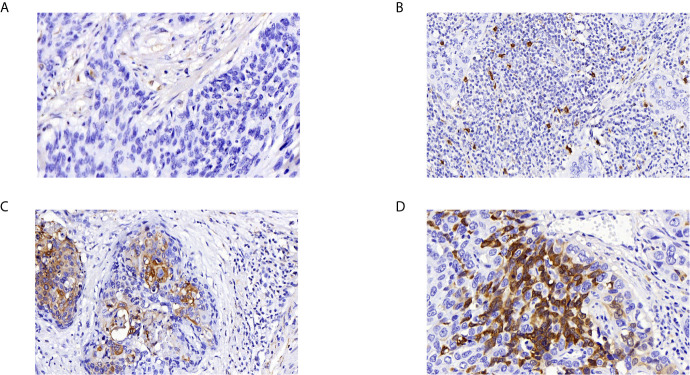
Immunohistochemical analysis of ADGRG1 expression in human cervical cancer specimens. **(A**–**D)** respectively represents negative, weak, moderate, strong intensity (magnifications, 400×).

We analyzed 132 paraffin‐embedded cervical squamous carcinoma tissues by immunohistochemistry. As shown in **Figure 4**, ADGRG1 was mainly located in the cytoplasm. **Figures 4A–D** respectively showed negative, weakly positive, moderately positive, and strongly positive expression intensity of cervical cancer slices. According to the scores of all samples, the 132 cases were divided into two groups (high expression, n = 68; low expression, n = 64). The correlation between ADGRG1 and clinicopathological parameters is summarized in **Table 1**. As a result, the expression level of ADGRG1 was significantly associated with FIGO stage (P = 0.0455), lymph node metastasis (P = 0.0426), depth of interstitial infiltration (P = 0.0140). Other parameters including age (P = 0.3595) and histological type (P = 0.1558) showed no statistical significance.

**Table 1:** Correlations between ADGRG1 expression and clinicopathologic characteristics in cervical cancer.

Characteristic	Cases	ADGRG1 expression		P
		Low expression	High expression	
Age				
≤50	87 (65.91)	45 (51.72)	42 (48.28)	0.3595
>50	45 (34.09)	19 (42.22)	26 (57.78)	
FIGO stage			
I	85 (64.39)	47 (55.29)	38 (44.71)	0.0455
II	47 (35.61)	17 (36.17)	30 (63.83)	
Histological grade			
Well/moderate	52 (39.39)	21 (40.38)	31 (59.62)	0.1558
Poorly	80 (60.61)	43 (53.75)	37 (46.25)	
Invasive interstatial depth			
<1/2	32 (24.24)	22 (68.75)	10 (31.25)	0.0140
≥1/2	100 (75.76)	42 (42.00)	58 (58.00)	
Lymphatic matastasis			
No	101 (76.52)	54 (53.47)	47 (46.53)	0.0426
Yes	31 (23.48)	10 (32.26)	21 (67.74)	

### ADGRG1 Expression in Cervical Cell Lines and Establishment of ADGRG1 Knockdown Cell Line

**Figure 5:**
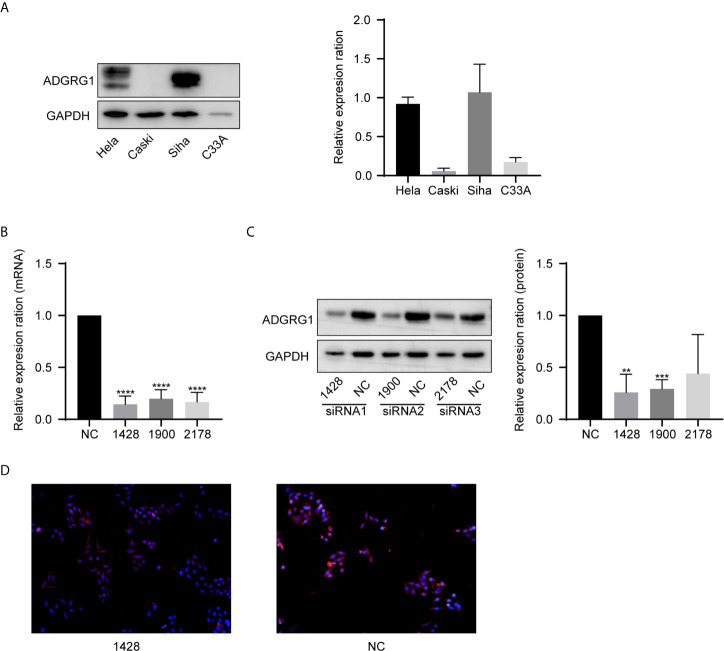
ADGRG1 expression in cell lines and construction of ADGRG knockdown cell line. **(A)** ADGRG1 protein expression in Hela, Siha, Caski, C33A by western blotting. **(B, C)** Expression of ADGRG1 was detected by qRT PCR and western blotting in Siha transfected with siRNA targeting GPR56 (si-1428, si-1900,si-2178) and negative control (si NC) (**P < 0.01, ***P < 0.001, ****P < 0.0001). **(D)** ADGRG1 knockdown effect shown by immunofluorescent images (magnifications, 200×).

We examined ADGRG1 expression levels in cervical cancer cell lines by western blotting, including Hela, Caski, Siha, C33A. The expressions of ADGRG1 in cervical cancer cell lines Siha and Hela were significantly higher than those in Caski and C33A at the protein level (**Figure 5A**). Then, we silenced ADGRG1 by transfecting Siha with specific siRNA (si−RNA1, si−RNA2, si−RNA3). The knockdown efficiency was detected by PCR (**Figure 5B**) and western blotting (**Figure 5C**). Immuno- fluorescence assay showed similar results (**Figure 5D**). Since the interference efficiency of si−RNA1 was significantly higher than those of si−RNA2 and si−RNA3, the si−RNA1 sequence was packaged into lentiviral vectors to construct the stable knockdown cell (Siha-KD) to perform the subsequent experiments.

### The Role of ADGRG1 in Cell Proliferation, Migration, Invasion, and Sensitivity to Cisplatin *In Vitro* and Relevant Mechanism

**Figure 6:**
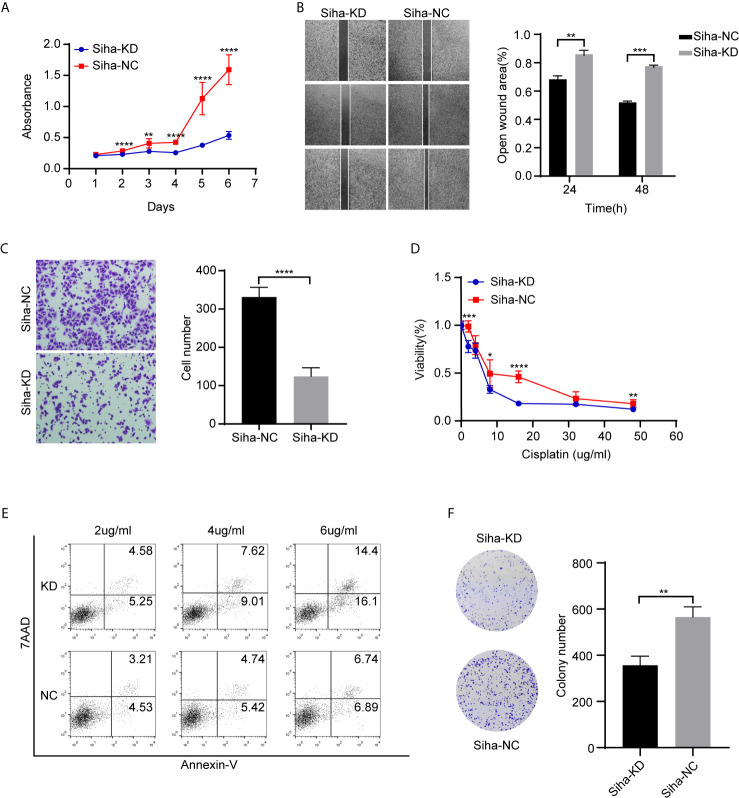
Effects of ADGR1 expression on cell proliferation, migration, invasion, and sensitivity to cisplatin. **(A)** Siha-KD exhibited decreased proliferation by CCK8 assay (**P < 0.01, ****P < 0.0001). **(B, C)** Downregulation of ADGRG1 inhibited cell migration and invasion by wound healing and transwell assays (**P < 0.01, ***P < 0.001, ****P < 0.0001). **(D)** Cell viability of Siha-KD was lower than Siha-NC when exposed to cisplatin (*P < 0.05, ***P < 0.001, ****P < 0.0001). **(E)** Knockdown of ADGRG1 enhanced cell apoptosis in cisplatin. **(F)** Siha-KD formed fewer colonies than Siha-NC in 2 μg/ml concentration of cisplatin. (**P < 0.01).

We performed Cell Counting Kit−8 (CCK−8) assays to detect the function of ADGRG1 in proliferation. In comparison to Siha-NC, Siha-KD exhibited decreased proliferation (**Figure 6A**). The wound scratch assay revealed that the silencing of ADGRG1 significantly suppressed cell migration (**Figure 6B**). The cell-invasive ability was seen decreased in Siha-KD by transwell matrigel-invasion assays (**Figure 6C**). Collectively, these results provided evidence that ADGRG1 plays vital roles in maintaining tumorigenicity *in vitro*. We tested the sensitivity of Siha-NC and Siha-KD towards cisplatin at gradient concentration by CCK-8 assay (**Figure 6D**) and cell apoptosis assay (**Figure 6E**). **Figure 6F** showed that Siha-KD formed fewer colonies in cisplatin of 2 ug/ml compared with Siha-NC. In a word, these three assays showed that the viability of Siha-KD was reduced compared with that of Siha-NC upon exposure to cisplatin.

We searched GEO with the keyword “ADGRG1.” GSE104653 was a microarray analysis of ADGRG1-knockout *versus* parental cells to identify gene expression changes upon ADGRG1 knockout in glioblastoma.

**Figure 7:**
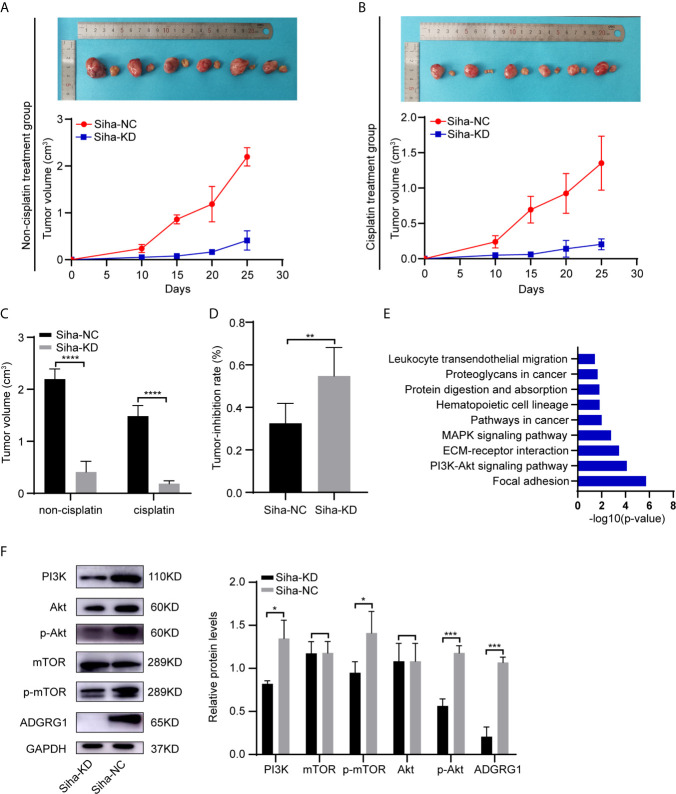
*In vivo* effects of ADGRG1 expression on tumorigenesis and sensitivity to cisplatin treatment, as well as relevant mechanism. **(A, B)** Photographs of xenograft tumors of Siha-NC and Siha-KD in non-cisplatin treatment group and cisplatin treatment group. **(C, D)** Growth curves of xenograft tumors in non-cisplatin treatment group and cisplatin treatment group (**P < 0.01, ****P < 0.0001). **(E)** KEGG pathway enrichment analysis of the DEGs from GSE104653 datasets. **(F)** Detection of PI3K/Akt signaling pathway-related genes at the protein level in Siha-KD and Siha-NC by western blotting (*p < 0.05, ***p < 0.001).

By performing GEO2R and DAVID analysis of GSE104653, we got the results of KEGG pathway enrichment. The pathways significantly enriched are shown as a bar chart in **Figure 7E**: Focal adhesion, PI3K-Akt signaling pathway, ECM-receptor interaction, MAPK signaling pathway, Pathways in cancer, Hematopoietic cell lineage, Protein digestion and absorption, Proteoglycans in cancer, Leukocyte transendothelial migration.

Additionally, western blot analysis assay showed that downregulation of ADGRG1 remarkably decreased the protein levels of PI3K, p-Akt, p-mTOR but not that of non−phosphorylated Akt and mTOR compared with the negative control Siha (**Figure 7F**). To summarize, knockdown of ADGRG1 decreases tumorigenesis *via* blockade of the P13K/Akt/mTOR axis in cervical cancer.

### Knockdown of ADGRG1 Suppresses Tumor Growth and Increases Sensitivity to Cisplatin *In Vivo*

To determine whether the silencing of ADGRG1 in cervical cancer could reduce tumor growth *in vivo*, SiHa cells transfected with negative control and ADGRG1-shRNA lentivirus were injected subcutaneously into BALB/c nude mice. As seen in **Figures 7A, C**, Siha-KD tumors grew at a slower rate compared with Siha-NC tumors. In the cisplatin treatment group, Siha-KD, which has a higher tumor reduction rate, appeared more sensitive to cisplatin (**Figures 7B, D**). All these data suggested that the inhibition of ADGRG1 in SiHa suppressed tumor growth, but improved its sensitivity and reactivity to cisplatin *in vivo*.

## Discussion

Owing to highly effective primary (HPV vaccine) and secondary (screening) prevention steps, incidence and mortality rates of cervical cancer have declined in most areas of the world for decades. Cervical cancer is still the fourth most frequently diagnosed carcinoma and the fourth leading cause of cancer death in women. Approximately 604,000 new cases and 342,000 deaths were expected in 2020 worldwide (23). The primary treatment of early-stage cervical cancer is either surgery or radiotherapy. Concurrent chemoradiation using platinum-containing chemotherapy is generally the primary treatment for IB3 to IVA disease, as well as for patients who are not suitable for hysterectomy. With single-agent cisplatin acting as a radiosensitizing agent, concurrent chemoradiation is superior to radiation alone (5, 24–27). Cisplatin-containing combination chemotherapy regimens have been considered the most effective agents for extrapelvic metastases and recurrent cases (28). However, cisplatin resistance is an obstacle to getting a satisfying outcome.

Cisplatin resistance is a multifactorial process referring to various mechanisms. The mechanisms include reduced intracellular accumulation of cisplatin, increased levels of DNA-damage repair, complex signal transduction network, cancer stem cell theory, metabolism, and energy (29–31). In our research, we induced cisplatin-resistant cervical squamous cancer cells by stimulation of gradient concentration cisplatin. Maybe the high-dose cisplatin stimulating method coincides highly with the mode of clinical chemotherapy, but the gradient concentration of the cisplatin can obtain higher and more stable resistance. To explore the molecular mechanisms of acquired cisplatin resistance in cervical squamous cell carcinoma, we obtained DEGs from RNA-sequencing of Siha-DDP and Siha-N. Simultaneously, we got DEGs in tissues of locally advanced squamous cervical cancer with poor responses to chemoradiotherapy or chemotherapy containing cisplatin from the GEO database. Because cisplatin treatment was the intersection of these three databases, overlapped genes ANK3 and ADGRG1 were most likely to be associated with cisplatin resistance. Based on the expression profile from GEPIA, ADGRG1 was overexpressed in cervical carcinoma compared with normal cervix uteri. However, there was no significant difference in the expression level of ANK3 between cervical cancer and adjacent normal tissues. Additionally, high expression of ADGRG1 predicts poor survival. These results suggest that ADGRG1 may play roles in tumorigenesis and cisplatin resistance. Therefore, we chose ADGRG1 as the target gene for further study.

ADGRG1 is a member of the ADGRG subfamily of aGPCRs (32). It is involved in the development of normal tissues and even in tumorigenesis (33). ADGRG1 is essential for the frontal cortex development in which function-deficient mutations lead to a severe malformation called bilateral frontoparietal polymicrogyria (BFPP) (34). It also participates in other developmental processes of muscle differentiation and immune regulation (15, 35). ADGRG1 even plays opposite roles in different tumors; for example, it serves as a suppressor in melanoma but possesses oncogenic property in glioblastoma, ovarian cancer, colon cancer, pancreatic cancer, non-small-cell lung cancer, and esophageal cancer (36). Nevertheless, the biological function of ADGRG1 remains poorly understood in cervical cancer.

Our study found that ADGRG1 was upregulated in cervical cancer tissues compared to the adjacent normal cervix. High expression was correlated to FIGO stage, invasive interstitial depth, and lymphatic metastasis, as well as poor progression-free survival. Cytological results indicated that knockdown of ADGRG1 gives rise to inhibition of cell proliferation, migration, and invasion. Its downregulation also enhances cell sensitivity to cisplatin. Subcutaneous xenograft assay also verified its function *in vivo*.

To explore the mechanism of ADGRG1 downregulation on tumorigenesis, we searched GEO with the keyword “ADGRG1.” GSE104653 was a microarray analysis of ADGRG1-knockout *versus* parental cells to identify gene expression changes upon ADGRG1 knockout in glioblastoma. Using GEO2R and DAVID, we got the results of KEGG pathway enrichment. The following pathways were significantly enriched: Focal adhesion, PI3K-Akt signaling pathway, ECM-receptor interaction, MAPK signaling pathway, Pathways in cancer, Hematopoietic cell lineage, Protein digestion and absorption, Proteoglycans in cancer, Leukocyte transendothelial migration. So we evaluated the PI3K/Akt signaling pathway expression in Siha-NC and Siha-KD by western blotting. The results demonstrated that PI3K, p-Akt, p-mTOR were downregulated in ADGRG1 knockdown group without inhibition of total Akt and mTOR. Thus, we inferred that knockdown of ADGRG1 might suppress cell proliferation, migration, and invasion and enhance sensitivity to cisplatin by inhibiting PI3K/Akt/mTOR pathway. In conclusion, ADGRG1 mediates tumor-invasive growth and chemoresistance *via* PI3K/Akt/mTOR signaling pathway.

## Data Availability Statement

The datasets presented in this study can be found in online repositories. The names of the repository/repositories and accession number(s) can be found in the article/supplementary material.

## Ethics Statement

The studies involving human participants were reviewed and approved by Ethics Committee of Qilu Hospital of Shandong University. The patients/participants provided their written informed consent to participate in this study. The animal study was reviewed and approved by Ethics Committee of Qilu Hospital of Shandong University.

## Author Contributions

XY and SZ designed the research. SZ performed the experiment. KG and KW collected data and performed statistical analysis. SZ and YL wrote the draft. SL revised the manuscript. All authors contributed to the article and approved the submitted version.

## Funding

The research was financially supported by the National Natural Science Foundation of China (No.81874105). National Natural Science Foundation of Shandong Province (No.ZR2016HQ24).

## Conflict of Interest

The authors declare that the research was conducted in the absence of any commercial or financial relationships that could be construed as a potential conflict of interest.

## References

[1] Arbyn M Weiderpass E Bruni L de Sanjosé S Saraiya M Ferlay J. Estimates of Incidence and Mortality of Cervical Cancer in 2018: A Worldwide Analysis. Lancet Glob Health (2020) 8(2):e191–203. 10.1016/s2214-109x(19)30482-6 PMC7025157 31812369

[2] Wei X Zhou Y Qiu J Wang X Xia Y Sui L. Low Expression of TUG1 Promotes Cisplatin Sensitivity in Cervical Cancer by Activating the MAPK Pathway. J buon (2019) 24(3):1020–6. 31424656

[3] Srinivasan R. Cervical Cancer Genomics: An Initial Step Towards Personalized Approach to Therapy. EBioMedicine (2019) 43:11–2. 10.1016/j.ebiom.2019.04.025 PMC6557915 31005518

[4] Thigpen T Shingleton H Homesley H Lagasse L Blessing J. Cis-Platinum in Treatment of Advanced or Recurrent Squamous Cell Carcinoma of the Cervix: A Phase II Study of the Gynecologic Oncology Group. Cancer (1981) 48(4):899–903. 10.1002/1097-0142(19810815)48:4<899::aid-cncr2820480406>3.0.co;2-6 7196794

[5] Rose PG Bundy BN Watkins EB Thigpen JT Deppe G Maiman MA. Concurrent Cisplatin-Based Radiotherapy and Chemotherapy for Locally Advanced Cervical Cancer. N Engl J Med (1999) 340(15):1144–53. 10.1056/nejm199904153401502 10202165

[6] Masadah R Rauf S Pratama MY Tiribelli C Pascut D. The Role of microRNAs in the Cisplatin- and Radio-Resistance of Cervical Cancer. Cancers (Basel) (2021) 13(5):1168–83. 10.3390/cancers13051168 PMC7963155 33803151

[7] Aoki Y Ochiai K Lim S Aoki D Kamiura S Lin H. Phase III Study of Cisplatin With or Without S-1 in Patients With Stage IVB, Recurrent, or Persistent Cervical Cancer. Br J Cancer (2018) 119(5):530–7. 10.1038/s41416-018-0206-7 PMC6162273 30072745

[8] Fennell DA Summers Y Cadranel J Benepal T Christoph DC Lal R. Cisplatin in the Modern Era: The Backbone of First-Line Chemotherapy for Non-Small Cell Lung Cancer. Cancer Treat Rev (2016) 44:42–50. 10.1016/j.ctrv.2016.01.003 26866673

[9] Fredriksson R Lagerström MC Lundin LG Schiöth HB. The G-Protein-Coupled Receptors in the Human Genome Form Five Main Families. Phylogenetic Analysis, Paralogon Groups, and Fingerprints. Mol Pharmacol (2003) 63(6):1256–72. 10.1124/mol.63.6.1256 12761335

[10] Lin HH. Adhesion Family of G Protein-Coupled Receptors and Cancer. Chang Gung Med J (2012) 35(1):15–27. 10.4103/2319-4170.106170 22483424

[11] Giera S Deng Y Luo R Ackerman SD Mogha A Monk KR. The Adhesion G Protein-Coupled Receptor GPR56 Is a Cell-Autonomous Regulator of Oligodendrocyte Development. Nat Commun (2015) 6:6121. 10.1038/ncomms7121 25607655 PMC4302951

[12] Piao X Chang BS Bodell A Woods K Benzeev B Topcu M. Genotype-Phenotype Analysis of Human Frontoparietal Polymicrogyria Syndromes. Ann Neurol (2005) 58(5):680–7. 10.1002/ana.20616 16240336

[13] Saito Y Kaneda K Suekane A Ichihara E Nakahata S Yamakawa N. Maintenance of the Hematopoietic Stem Cell Pool in Bone Marrow Niches by EVI1-Regulated GPR56. Leukemia (2013) 27(8):1637–49. 10.1038/leu.2013.75 23478665

[14] Chen G Yang L Begum S Xu L. GPR56 Is Essential for Testis Development and Male Fertility in Mice. Dev Dyn (2010) 239(12):3358–67. 10.1002/dvdy.22468 PMC2991479 20981830

[15] Wu MP Doyle JR Barry B Beauvais A Rozkalne A Piao X. G-Protein Coupled Receptor 56 Promotes Myoblast Fusion Through Serum Response Factor- and Nuclear Factor of Activated T-Cell-Mediated Signalling But Is Not Essential for Muscle Development *In Vivo* . FEBS J (2013) 280(23):6097–113. 10.1111/febs.12529 PMC3877849 24102982

[16] White JP Wrann CD Rao RR Nair SK Jedrychowski MP You JS. G Protein-Coupled Receptor 56 Regulates Mechanical Overload-Induced Muscle Hypertrophy. Proc Natl Acad Sci USA (2014) 111(44):15756–61. 10.1073/pnas.1417898111 PMC4226111 25336758

[17] Daria D Kirsten N Muranyi A Mulaw M Ihme S Kechter A. GPR56 Contributes to the Development of Acute Myeloid Leukemia in Mice. Leukemia (2016) 30(8):1734–41. 10.1038/leu.2016.76 27063597

[18] Huang Y Fan J Yang J Zhu GZ. Characterization of GPR56 Protein and Its Suppressed Expression in Human Pancreatic Cancer Cells. Mol Cell Biochem (2008) 308(1-2):133–9. 10.1007/s11010-007-9621-4 17932623

[19] Zhang S Chatterjee T Godoy C Wu L Liu QJ Carmon KS. GPR56 Drives Colorectal Tumor Growth and Promotes Drug Resistance Through Upregulation of MDR1 Expression *via* a RhoA-Mediated Mechanism. Mol Cancer Res (2019) 17(11):2196–207. 10.1158/1541-7786.Mcr-19-0436 PMC6913243 31444231

[20] Liu Z Huang Z Yang W Li Z Xing S Li H. Expression of Orphan GPR56 Correlates With Tumor Progression in Human Epithelial Ovarian Cancer. Neoplasma (2017) 64(1):32–9. 10.4149/neo_2017_104 27881002

[21] Shashidhar S Lorente G Nagavarapu U Nelson A Kuo J Cummins J. GPR56 Is a GPCR That Is Overexpressed in Gliomas and Functions in Tumor Cell Adhesion. Oncogene (2005) 24(10):1673–82. 10.1038/sj.onc.1208395 15674329

[22] Yang L Friedland S Corson N Xu L. GPR56 Inhibits Melanoma Growth by Internalizing and Degrading Its Ligand TG2. Cancer Res (2014) 74(4):1022–31. 10.1158/0008-5472.Can-13-1268 PMC3944670 24356421

[23] Sung H Ferlay J Siegel RL Laversanne M Soerjomataram I Jemal A. Global Cancer Statistics 2020: GLOBOCAN Estimates of Incidence and Mortality Worldwide for 36 Cancers in 185 Countries. CA Cancer J Clin (2021) 71(3):209–49. 10.3322/caac.21660 33538338

[24] Whitney CW Sause W Bundy BN Malfetano JH Hannigan EV Fowler WC Jr. Randomized Comparison of Fluorouracil Plus Cisplatin Versus Hydroxyurea as an Adjunct to Radiation Therapy in Stage IIB-IVA Carcinoma of the Cervix With Negative Para-Aortic Lymph Nodes: A Gynecologic Oncology Group and Southwest Oncology Group Study. J Clin Oncol (1999) 17(5):1339–48. 10.1200/jco.1999.17.5.1339 10334517

[25] Morris M Eifel PJ Lu J Grigsby PW Levenback C Stevens RE. Pelvic Radiation With Concurrent Chemotherapy Compared With Pelvic and Para-Aortic Radiation for High-Risk Cervical Cancer. N Engl J Med (1999) 340(15):1137–43. 10.1056/nejm199904153401501 10202164

[26] Peters WA 3rd Liu PY Barrett RJ 2nd Stock RJ Monk BJ Berek JS. Concurrent Chemotherapy and Pelvic Radiation Therapy Compared With Pelvic Radiation Therapy Alone as Adjuvant Therapy After Radical Surgery in High-Risk Early-Stage Cancer of the Cervix. J Clin Oncol (2000) 18(8):1606–13. 10.1200/jco.2000.18.8.1606 10764420

[27] Keys HM Bundy BN Stehman FB Muderspach LI Chafe WE Suggs CL 3rd. Cisplatin, Radiation, and Adjuvant Hysterectomy Compared With Radiation and Adjuvant Hysterectomy for Bulky Stage IB Cervical Carcinoma. N Engl J Med (1999) 340(15):1154–61. 10.1056/nejm199904153401503 10202166

[28] Moore DH Blessing JA McQuellon RP Thaler HT Cella D Benda J. Phase III Study of Cisplatin With or Without Paclitaxel in Stage IVB, Recurrent, or Persistent Squamous Cell Carcinoma of the Cervix: A Gynecologic Oncology Group Study. J Clin Oncol (2004) 22(15):3113–9. 10.1200/jco.2004.04.170 15284262

[29] Galluzzi L Senovilla L Vitale I Michels J Martins I Kepp O. Molecular Mechanisms of Cisplatin Resistance. Oncogene (2012) 31(15):1869–83. 10.1038/onc.2011.384 21892204

[30] Shen DW Pouliot LM Hall MD Gottesman MM. Cisplatin Resistance: A Cellular Self-Defense Mechanism Resulting From Multiple Epigenetic and Genetic Changes. Pharmacol Rev (2012) 64(3):706–21. 10.1124/pr.111.005637 PMC3400836 22659329

[31] Ferreira JA Peixoto A Neves M Gaiteiro C Reis CA Assaraf YG. Mechanisms of Cisplatin Resistance and Targeting of Cancer Stem Cells: Adding Glycosylation to the Equation. Drug Resist Update (2016) 24:34–54. 10.1016/j.drup.2015.11.003 26830314

[32] Hamann J Aust G Araç D Engel FB Formstone C Fredriksson R. International Union of Basic and Clinical Pharmacology. XCIV. Adhesion G Protein-Coupled Receptors. Pharmacol Rev (2015) 67(2):338–67. 10.1124/pr.114.009647 PMC4394687 25713288

[33] Huang KY Lin HH. The Activation and Signaling Mechanisms of GPR56/ADGRG1 in Melanoma Cell. Front Oncol (2018) 8:304. 10.3389/fonc.2018.00304 30135857 PMC6092491

[34] Piao X Hill RS Bodell A Chang BS Basel-Vanagaite L Straussberg R. G Protein-Coupled Receptor-Dependent Development of Human Frontal Cortex. Science (2004) 303(5666):2033–6. 10.1126/science.1092780 15044805

[35] Chang GW Hsiao CC Peng YM Vieira Braga FA Kragten NA Remmerswaal EB. The Adhesion G Protein-Coupled Receptor GPR56/ADGRG1 Is an Inhibitory Receptor on Human NK Cells. Cell Rep (2016) 15(8):1757–70. 10.1016/j.celrep.2016.04.053 27184850

[36] Jin Z Luo R Piao X. GPR56 and Its Related Diseases. Prog Mol Biol Transl Sci (2009) 89:1–13. 10.1016/s1877-1173(09)89001-7 20374731

